# THREE INNOVATIVE DATA RESOURCES: EDSHARE, THE MULTIGENERATIONAL LONGITUDINAL PANEL, AND THE 1940 CENSUS

**DOI:** 10.1093/geroni/igae098.2077

**Published:** 2024-12-31

**Authors:** John Warren

**Affiliations:** University of Minnesota, Minneapolis, Minnesota, United States

## Abstract

Scientists interested in studying the long-term health consequences of early life social, economic, policy, environmental, or other exposures have historically suffered from a lack of suitable data. Large, nationally representative studies of early life (e.g., Add Health) rarely extend late enough into older ages. At the same time, large, nationally representative studies of later life (e.g., HRS, NSHAP) rarely contain prospectively gathered, high resolution information about early life exposures. This talk will describe three new and extraordinary new data resources that overcome these problems. FIRST, the Education Studies for Health Aging Research (EdSHARe) project has re-purposed two large national studies of adolescence and education started in the 1970s and 1980 to be studies of health and aging; the talk will focus on these cohort studies utility for understanding the social and biological pathways through which early life factors --- especially education --- impact later life health and cognition. SECOND, the Multigenerational Longitudinal Panel (MLP) includes linked complete-count U.S. census records for every American from 1850 through 2020 plus other mortality, vital records, and other kinds of data. THIRD, as an offshoot of MLP, we have linked records about older people from the HRS, NSHAP, PSID, WLS, and NHATS to the 1940 Census in which aging survey participants are seen in childhood along with their parents and other family members. All three data resources are or soon will be available to the research community; all stand to transform aging research.

